# Depletion reveals role of bicarbonate in the photosynthetic electron transport chain of *Limnospira maxima*

**DOI:** 10.3389/fpls.2025.1584909

**Published:** 2025-06-16

**Authors:** Leslie Castillo, Stavroula Nicolaou, Colin Gates

**Affiliations:** ^1^ Department of Chemistry and Biochemistry, Loyola University Chicago, Chicago, IL, United States; ^2^ Department of Biology, Loyola University Chicago, Chicago, IL, United States; ^3^ Department of Bioinformatics, Loyola University Chicago, Chicago, IL, United States

**Keywords:** photosystem II, bicarbonate, non-heme iron, plastoquinone, photosynthetic electron transport chain, arthrospira maxima

## Abstract

Efficient photosynthetic light reactions require tight balancing of electrons and protons. In photosystem II, bicarbonate is coordinated to a non-heme iron positioned between the acceptor-side plastoquinones Q_A_ and Q_B_, modulating electron transfer. The hypercarbonate-requiring filamentous cyanobacterium *Limnospira maxima* has had multiple bicarbonate functions on both acceptor and donor side of PSII determined by depletion. 77K spectrofluorometric investigation of phycobilin and chlorophyll excitation suggests the mild *in vivo* depletion method for bicarbonate results in loss of chlorophyll connectivity to the reaction center in PSII and dissociation of the phycobilisome due to proportional increase of fluorescence emission from allophycocyanin. Using chlorophyll fast repetition rate fluorometry, it was observed under bicarbonate depletion that oscillations were still present in a fraction of PSIIs, confirming the functionality of the water oxidizing complex remains in this fraction of centers. In these fractions of centers only one to two electrons are being released. Q_A_^-^ reoxidation kinetics indicate that loss of bicarbonate permits successful electron transfer to Q_B_, forming semiquinone Q_B_^-^. DCMU inhibition of the second electron transfer implies that, in bicarbonate-depleted *L. maxima*, electron transfer to Q_B_ affects proton delivery needed for plastoquinol formation, which suggests that this depletion targets the non-heme iron. Based on cytochrome b_6_f redox kinetics, depleted cells experience less intensity of oxidation and upon illumination cytochrome b and f are proportionally rapidly and intensely oxidized. P700 redox kinetics exhibit a delay feature in PSI as well as the expected delay of electron delivery to PSII, suggesting a further bicarbonate effect on PSI.

## 1 Introduction

All living organisms, from bacteria to humans, require energy input to sustain life. Oxygenic photosynthesis is the most widespread and successful process for producing biologically available energy on Earth (Payne et al., 2011). It is responsible for nearly all molecular oxygen in our atmosphere and fixed carbon in the biosphere. In oxygenic phototrophs like cyanobacteria, algae, and higher plants, electrons necessary for this process are derived from water. The initiation of photosynthetic electron transport begins with photosystem II (PSII), an enzyme complex that is highly conserved across the tree of life (Shen, 2015). Within PSII, light energy is absorbed by antenna pigments and transferred to the reaction center, where charge separation occurs and electrons are extracted from water. PSII facilitates the conversion of light energy into chemical energy by driving water oxidation and plastoquinone reduction, resulting in the production of biologically available reductant, proton motive force, and ATP. Photosynthetic water oxidation takes place at the water oxidizing complex (WOC), the Mn_4_CaO_5_ cluster which is surrounded by “water channels” that facilitate the inflow of water and the outflow of protons and oxygen (Vinyard and Brudvig, 2017; Kaur et al., 2021). Oxygen production is achieved with the sequential accumulation of four charges through a cycle of 4 semi-stable to stable intermediate oxidation states (S-states) and one transient one (S_4_) (Vinyard et al., 2013a). Transitions from S_0_ to S_3_ involve charge separation events leading to the removal of four total electrons to tyrosine-Z (Y_z_) and from there to the reaction center of PSII, the special chlorophyll P_680_. These transitions in total release four protons and one oxygen molecule per cycle, and lead to four reductions of downstream cofactors where, ultimately, plastoquinone (PQ) is reduced to plastoquinol (PQH_2_) on the acceptor side (Vinyard et al., 2013a; Nilsson et al., 2016).

Photosynthetic organisms rely on atmospheric carbon dioxide as the terminal acceptor to store sunlight energy in carbohydrates (Junge, 2019). Oxygenic phototrophs additionally require CO_2_ in solution, typically in the form of carbonic acid (H_2_CO_3_) and bicarbonate ions (HCO_3_^-^), for optimal function of PSII, which is known as the “bicarbonate effect” (Stemler, 2002). Bicarbonate ions regulate electron transfer within PSII in several ways (Gates et al., 2023a). Developments in structural biology have provided detailed insights into the molecular structure of PSII, including specific binding sites. Studies confirmed that PSII binds one bicarbonate molecule as a ligand to the non-heme iron (NHI) on the acceptor side (Tikhonov et al., 2018), while bicarbonate optimizes the water-splitting reactions on the donor side (Shevela et al., 2012). Bicarbonate directly plays a variety of regulatory roles in photosynthetic operation, influencing electron flow at both donor and acceptor side (Blubaugh and Govindjee, 1988; Van Rensen and Xu, 1999; Stemler, 2002; McConnell et al., 2012; Shevela et al., 2012). At the donor side, bicarbonate interacts with small lumenal subunits that aid in the photoprotection of the WOC (Klimov and Baranov, 2001; Tikhonov et al., 2018; Vinyard and Govindjee, 2024). It is also believed to act as a proton transfer mediator during water splitting (Ulas and Brudvig, 2010; Banerjee et al., 2019; Gates et al., 2023b). Furthermore, bicarbonate ions located in the lumenal space have been shown to be essential for photoassembly of the Mn_4_CaO_5_ cluster via pairing with positively charged residues that result in navigation of Mn^2+^ to the WOC site that accelerates the rate of WOC formation and increases the yield of O_2_ (Baranov et al., 2000; Dasgupta et al., 2008; Ananyev et al., 2018; Tikhonov et al., 2018; Vinyard and Govindjee, 2024; Shitov et al., 2025). On the acceptor side, bicarbonate is coordinated to the NHI positioned between the two integral plastoquinones Q_A_ and Q_B_ (Noguchi and Inoue, 1995; Cox et al., 2009; Saito et al., 2013; Sugo and Ishikita, 2022). This ligand impacts both electron transfer and acquisition of protons at Q_B_. Notably, bicarbonate is posited to be the terminal base for neutralization of protons released during water oxidation. This role was first proposed based on the operation of PSII in *Limnospira maxima (L. maxima) (* Ananyev and Dismukes, 2005; Ananyev et al., 2018).

To gain insights into the role of bicarbonate in PSII, depletion is used as a tool to observe how its absence affects biochemical reactions such as electron transfer steps. Prior studies have introduced novel techniques for removing dissolved inorganic carbon (DIC, often as bicarbonate) and investigating its impact on oxygen evolution of PSII (Ananyev et al., 2018). Bicarbonate chelators such as sodium formate, arginine, and magnesium chloride have been used as a method of removal from cells (Stemler, 1980; Hunger et al., 2013). Arginine residues are known to form strong ionic bonds with bicarbonate (Ananyev et al., 2005; Hwang et al., 2007; Armstrong et al., 2016). It was observed that depletion at the non-heme iron site and solvent-accessible arginines appears to significantly diminish O_2_ yield due to O_2_ uptake, while potentially expediting transitions in the water oxidation cycle. Two roles of bicarbonate on the acceptor side of *L. maxima* were hypothesized to exist with distinct depletion mechanisms but definite sites were not confirmed. Our aim is to determine those sites through depletion studies.

*Limnospira maxima* (formerly known as *Arthrospira maxima*) is a distinctive cyanobacterium that thrives in alkaline lakes at high pH of 10-11 (Vonshak and Tomaselli, 2002; Carrieri et al., 2007). It can grow in high concentrations of bicarbonate (up to 1.2M), which is a toxic environment for most organisms. Along with this robust tolerance, it has evolved a preference for bicarbonate uptake over CO_2_ (Carrieri et al., 2007; Ananyev et al., 2018). This elevated bicarbonate level is thought to contribute to unique behavior of its PSII acceptor side compared to other phototrophs (Ananyev and Dismukes, 2005; Ananyev et al., 2016; Ananyev et al., 2018). Most phototrophs exhibit limitations on the acceptor side due to turnover of the plastoquinone (PQ) pool, i.e. removal of electrons from Q_B_ to cytochrome b_6_f (Cyt b_6_f) (Shevela et al., 2023). However, limitations on the acceptor side have not been seen (the PQ pool turnover frequency is at least 500 Hz), and the rate limiting step in *L. maxima* is on the donor side (Ananyev and Dismukes, 2005; Ananyev et al., 2016; Colin Gates, 2017). Previous research has emphasized the heightened significance of this role, particularly highlighting its rapid electron transfer on the acceptor side. Moreover, studies have shown the high efficiency of its water oxidizing complex compared to other organisms (Ananyev and Dismukes, 2005; Gates et al., 2016). Given these interconnected traits, our aim is to assess the effects of depletion on both the donor and acceptor side, with a key focus on understanding why the acceptor side does not exhibit limitation.

## 2 Materials and methods

### 2.1 Cultures

*Limnospira maxima* (UTEX LB 2342) was obtained from the University of Texas at Austin Culture Collection and was incubated in a Caron growth chamber at 30°C in 75 cm^2^ culture flasks containing 100 mL of Zarrouk medium (Tarko et al., 2012). Cultures were illuminated under 25 µmol photons/m^2^/s continuous white light from Caron gBrite LED lamps at 12/12h dark/light cycles. Samples were taken in log phase (mid-exponential) growth (six to eight-day old culture); see **Supplementary Figure S1**. To measure the growth phase of samples, the optical density (OD_730_) was measured at 730 nm using a Thermo-Fisher Genesys 10 spectrophotometer.

### 2.2 Removal of bicarbonate

*Limnospira maxima* cells were depleted of bicarbonate via wash treatment by Zarrouk medium without bicarbonate and with 100 mM sodium formate (pH 7.8), serving as an isotonic buffer to prevent osmotic pressure imbalances in the cells. Sodium formate is structurally similar to bicarbonate but cannot dissociate to produce carbon dioxide and water or hydroxide. It also has a lower apparent pK_a_ for the transition to its conjugate acid due to the inability to readily interconvert with a gaseous form (bicarbonate/carbonic acid/carbon dioxide) (Hillier et al., 2006). Samples were centrifuged at 9,000 x *g* for 10 minutes each and resuspended. The samples were washed four times with the formate wash medium and the supernatant was discarded between each treatment (Ananyev et al., 2018). This method was performed to ensure a removal of bicarbonate within the cells. Samples were kept in darkness for two hours prior to testing. To reverse bicarbonate effects, NaHCO_3_ was reintroduced to the cells at their usual concentration (200 mM) by washing them four times in their native medium. Samples were washed under complete darkness and kept in the dark until otherwise specified.

### 2.3 Oximetry

The rate oxygen production of cultures was assessed using a Hansatech Oxygraph+ Clark-type oxygen electrode (Delieu and Walker, 1972). To measure oxygen consumption during respiration, cells were measured in the dark for five minutes with continuous stirring. To measure oxygen production under growth light, cells were exposed to 25 µmol photons/m^2^/s continuous white light (growth light) for five minutes. Results are based on technical replicates (n=10) from individual experiments and are representative of the observed trends.

### 2.4 Fluorescence emission spectroscopy

To observe the light harvesting and exciton usage of the cells, fluorescence emission was measured using a JASCO FP-8300 fluorometer with PMU-830 liquid nitrogen sample assembly. Measurements were taken at low temperatures of 77K (Lamb et al., 2018). Measurement at low temperatures is a common method used in photosynthesis research to distinguish fluorescence between photosystem I (PSI) and photosystem II (PSII) (Bruce et al., 1989; Andrizhiyevskaya et al., 2005). At low temperatures, unwanted physiological acclimations and biochemical reactions are eliminated and, specifically, proteins cannot associate or dissociate during measurements (Lamb et al., 2018). The two major fluorescence emission bands generated from 77K spectrofluorometry allow us to observe PSII and PSI. Samples were excited at optimal absorbance wavelengths by antenna pigment (chlorophyll 435 nm and phycobilin 561 nm) and emission was observed in the range 580 nm-750 nm. By using this method, we can determine changes in connectivity of antenna, balance, composition, and protein complex integrity between the two photosystems. Results are based on six technical replicates from individual experiments representative of three biological replicates and are consistent across all measured conditions.

### 2.5 Fast repetition rate fluorometry

Chlorophyll fluorescence measurements (Béal et al., 1999; Ananyev and Dismukes, 2005; Ananyev et al., 2018; Gates et al., 2020) were taken using a SpectroLogiX JTS-150 (Joliot-type) spectrometer modified to serve as a fast repetition rate (FRR) fluorometer. FRR allows for rapid (single-excitation) measurements of the kinetics of Chl *a* variable fluorescence yield (F_v_). The F_v_ measures the yield of P680 chlorophyll emission from only active PSII and (F_v_/F_m_) was determined from F_v_=F_m_-F_0_, where F_0_ is the initial fluorescence upon stimulation with light and F_m_ is the maximal, saturated fluorescence (Ananyev and Dismukes, 2005). Single turnover flash (STF; enough light to advance all WOC centers through one S-state transition, but delivered over a short enough time for further excitations to not be possible) oscillations were produced using a RPMC Lasers 5-watt TTL controlled laser with a bandwidth of 10 nm and centerpoint of 636.5 nm which generated pulses as single turnover flashes of pulse duration 4.5 µs. The oscillations of F_v_/F_m_ were analyzed and processed through a model-dependent nonlinear least-squares fitting using modeling software implementing the VZAD algorithm (Vinyard et al., 2013b). WOC cycle parameters and S-state population distributions were obtained from fitting the data to the VZAD model, which is a modified version of the Joliot-Kok model (Kok et al., 1970). The accuracy was confirmed by a root-mean-squares deviation of the normalized and experimental fit oscillations. All data was collected from the mean values of 15 trains of 50 flashes each and thus are averages of 15 technical replicates.

To measure Q_A_^-^ reoxidation, the same fluorometer was used. Dark-adapted (120 s) cells were subjected to a pair of pump-probe flashes with varying intervals between the pump and probe, determining the kinetics of return from maximum fluorescence (F_m_) at time zero (when all centers are saturated) to the initial fluorescence (F_o_) after sufficient time for complete Q_A_^-^ decay. The actinic flash used to induce Q_A_− formation was a single turnover flash with a duration of 24 µs and an intensity of approximately 470,000 µmol photons/m^2^/s. The resulting data were fitted to a biphasic exponential model, from which the parameters for Q_A_^-^ reoxidation were obtained (Gorbunov et al., 1999). For FRR measurements, results are representative of at least 4 individual technical replicates for all conditions. Similarly, for Q_A_^−^ reoxidation, results are also representative of at least 6 individual technical replicates.

### 2.6 Absorbance redox kinetics

Kinetics of the change in absorbance of redox-active species associated with cytochrome b_6_f were measured using the same SpectroLogiX JTS-150 spectrometer (Ungerer et al., 2018; Buchert et al., 2022). An LED actinic light was used to stimulate photosynthesis at 630 nm. Absorbance was measured at 546 nm (cytochrome b), 554 nm (cytochrome f), and 574 nm (plastocyanin) to calculate relative absorbance, and therefore concentration of reduced forms of heme in cytochrome b and cytochrome f and the Cu-S-imidazole active site of (reduced) plastocyanin. A multiple bandpass filter (BG-39) was used to allow concurrent absorbance measurements at all necessary wavelengths.

For the measurement of PSI redox kinetics, the absorbance of photooxidized P700 (the reaction center chlorophyll of PSI) was measured using the same spectrometer. A dedicated multiple-bandpass P700 filter was used, centered at 810 nm to monitor the appearance and decay of P700^+^ absorbance during the redox process. This wavelength corresponds to a near-infrared absorption band of oxidized P700, allowing for accurate tracking of its reduction kinetics. Additional transmission at 705 and 740 nm was included in the filter to assist with data confirmation and correction for scattering artifacts (Klughammer and Schreiber, 1994; Nguyen et al., 2017). Results represent the average of three technical replicates representative of three biological replicates for both cytochrome b_6_f and P700 measurements.

## 3 Results

### 3.1 Oximetry

By determining both oxygen consumption and net evolution the rate of original oxygen production from photosynthesis can be determined. For native *L. maxima*, the oxygen production was 164.14 µmol O_2_ mg chl−¹ h−¹ under a light intensity of 20 µmol photons/m^2^/s. To determine the effect of the bicarbonate depletion method on photosynthetic activity, we measured the oxygen production of depleted cells. Depletion led to a decrease of oxygen production to 69.41 µmol O_2_ mg chl−¹ h−¹, representing over 50% reduction. This affirmed the effectiveness of the depletion method. The reversal of bicarbonate (repletion) and oxygen production was also studied to see if this method was reversible and to assess potential damage to PSII. Depletion of bicarbonate is completely reversible as seen by the yield of 165.98 µmol O_2_ mg chl−¹ h−¹ after reversal, which is negligibly higher than in the untreated cells.

### 3.2 Fluorescence emission spectroscopy

**Figure 1:**
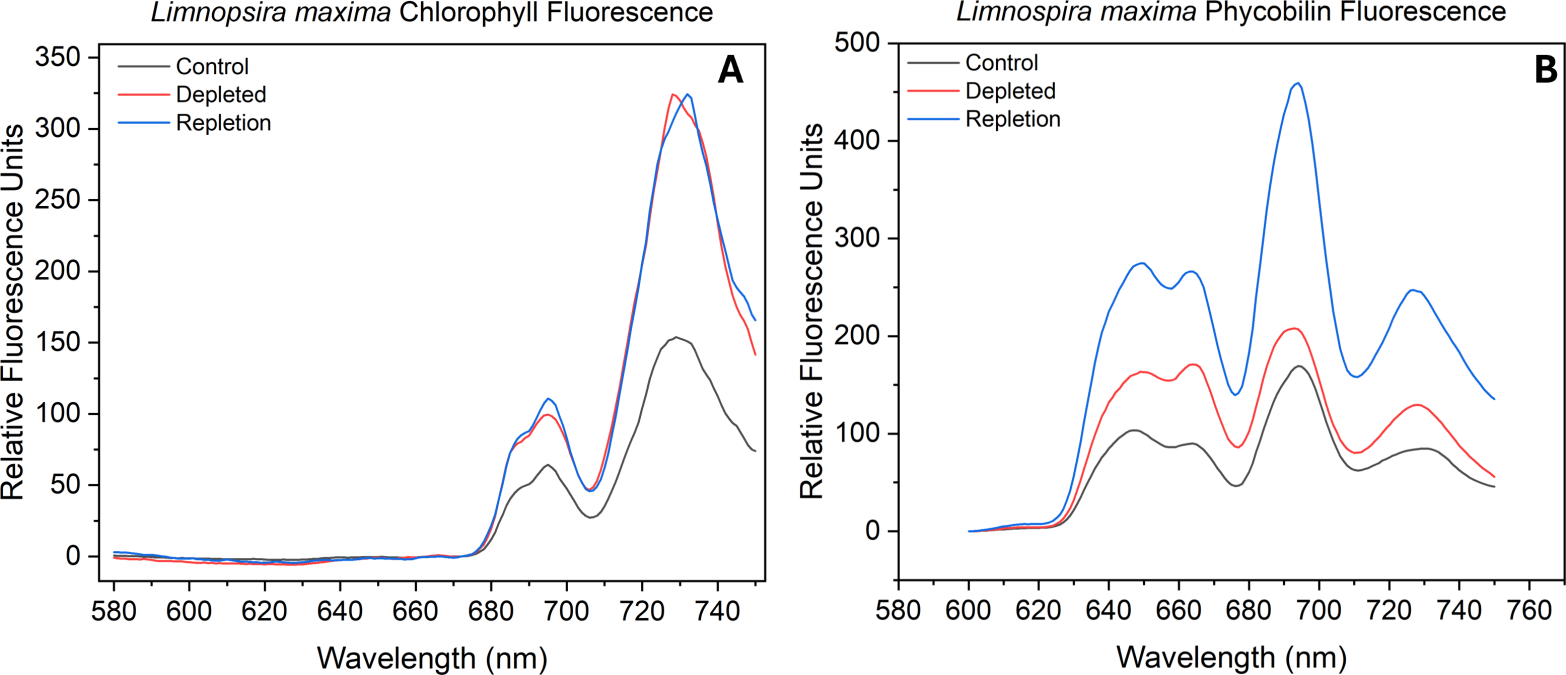
Fluorescence emission spectra of **(A)** chlorophyll and **(B)** phycobilin of control sample, bicarbonate depleted and after repletion of bicarbonate in *Limnospira maxima* at 77K. Samples were excited at desired wavelengths (chlorophyll at 435 nm and phycobilin at 561 nm).

Photosynthetic organisms have the ability to regulate distribution of excitation energy between PSII and PSI by modulating stoichiometry and attachment of antenna proteins (Bruce et al., 1989). Here we observed the fluorescence derived from excitation of two antenna pigments of interest: chlorophyll and phycobilin. The peak wavelengths of PSII and PSI were identified through direct excitation of photosystem-associated chlorophyll and the emission ratios of the two photosystems were determined along with the F685 (reaction center) and F695 (CP47 trap) subpeaks of PSII (Boehm et al., 2011; Hall et al., 2016). The fluorescence emission was normalized to the PSI maximum fluorescence emission peak for processing. **Figure 1A** displays the terminal exciton recipient fluorescence (chlorophyll) of control, depleted and bicarbonate repleted *L. maxima*. A red-shifted chlorophyll “traps” at 694–697 nm (F695), linked to PSII emission from centers unable to advance excitons to P680 (Andrizhiyevskaya et al., 2005). In depleted cells, the PSI emission peak is notably blue-shifted by approximately 1 nm relative to the control and repleted cells. In **Table 1**, a gaussian fit (**Supplementary Figures S2**-**S7**; **Supplementary Tables S2**-**S7**) shows the control yielded a F685:F695 ratio of 0.23 while the depleted sample had a higher yield of 0.38, suggesting more fluorescence from “healthier” PSII. The repleted sample had an even higher F685:F695 ratio of 0.45, exceeding both the control and depleted samples. The PSII: PSI ratio is monitored to determine any changes in the amount of PSII needed to stoichiometrically balance the electron transport chain’s operation. In the depleted cells, removal of bicarbonate causes a substantial effect on exciton distribution, shifting from a ratio of 0.27 (control) to 0.19 (depleted). This suggests there is more chlorophyll exciton delivery to the reaction center in PSII when bicarbonate is present compared to PSI. When bicarbonate is reintroduced to depleted cells, chlorophyll exciton delivery to P680 increases, reaching a PSII: PSI ratio of 0.21, much closer to the depleted cells.

**Table 1:** Emission peak ratios of: F685 and F695 and PSII: PSI from chlorophyll excitation (435 nm) at 77K.

Chlorophyll and Phycobilin Peak Area Ratios						
	Chlorophyll		Phycobilin			
	F685:F695	PSII: PSI	F685:F695	PSII: PSI	APC: CPC	PB : PS
Control	0.23	0.27	0.14	0.93	0.77	0.57
Depleted	0.38	0.19	0.12	1.00	0.88	0.74
Repletion	0.45	0.21	0.10	1.02	0.94	0.61

F685: F695, PSII : PSI, APC: CPC and PB : PS emission peak area ratios from phycobilin excitation (561 nm) at 77K emission ratio.

The F685:F695 ratio indicates a comparable “health” status of PSII in the depleted *L. maxima* cells (0.12) compared to the control (0.14), suggesting that the exciton backup is not significantly different in the absence of bicarbonate, as the antenna remains attached. Monitoring the fluorescence emission of F685:F695 can provide insights into the efficiency of energy transfer within PSII and the general health of the system. The repleted cells resulted in fluorescence emission ratios similar to that of the control; adding bicarbonate resulted in an F685:F695 ratio of 0.10. The PSII: PSI ratios can indicate where the phycobilin antenna is attached and whether it is associated more with PSII or PSI. The chlorophyll PSII: PSI ratios obtained were as follows: control *L. maxima* (0.93), bicarbonate depleted (1.00), and repletion of bicarbonate (1.02) (see **Table 1**). Bicarbonate depletion resulted in a slightly higher PSII: PSI ratio compared to the control but did not significantly affect PSII antenna modulation, as depleted sampled showed no notable change in antenna connectivity antenna connectivity to either photosystem.

Two distinct subpeaks are seen in the fluorescence emission of phycobilin (see **Figure 1B**). The leftmost peak, representing shorter wavelength and higher energy pigments, is notably higher and linked to c-phycocyanin (CPC). Absorption of light at a higher energy pigment is subsequently transferred to the lower energy pigment, allophycocyanin (APC), indicated by the adjacent right subpeak. Through measuring the excitation of these protein-pigment complexes, we can observe the balance of exciton flow by assessing the fluorescence emission from the phycobilin. As seen in **Table 1**, untreated *L. maxima* has a relatively low APC: CPC (0.77) ratio, while bicarbonate depleted cells have a higher APC: CPC (0.88). Native *L. maxima* shows a lower APC yield and more CPC, consistent with the higher abundance of CPC compared to APC. Repleted cells had an APC: CPC ratio of 0.94, higher than both control and depleted cells, indicating more efficient exciton transfer to the reaction center. Measuring phycobilin fluorescence emission can determine the ratio of free phycobilin (PB) to photosystems (PS), enabling further measurement of the distribution of the exciton path from the phycobilisome. When bicarbonate is present, the ratio of PB: PS is 0.57, while in its absence, the ratio is higher at 0.74. This suggests that some phycobilins are unable to forward excitons to a reaction center without bicarbonate. Repleted cells reverted to comparable fluorescence emission of the PB: PS of the control, with a ratio of 0.61. There is proportionally more phycobilin association with PSII and PSI as seen in the PSII: PSI ratio under bicarbonate conditions.

### 3.3 Chlorophyll variable fluorescence kinetics

**Figure 2:**
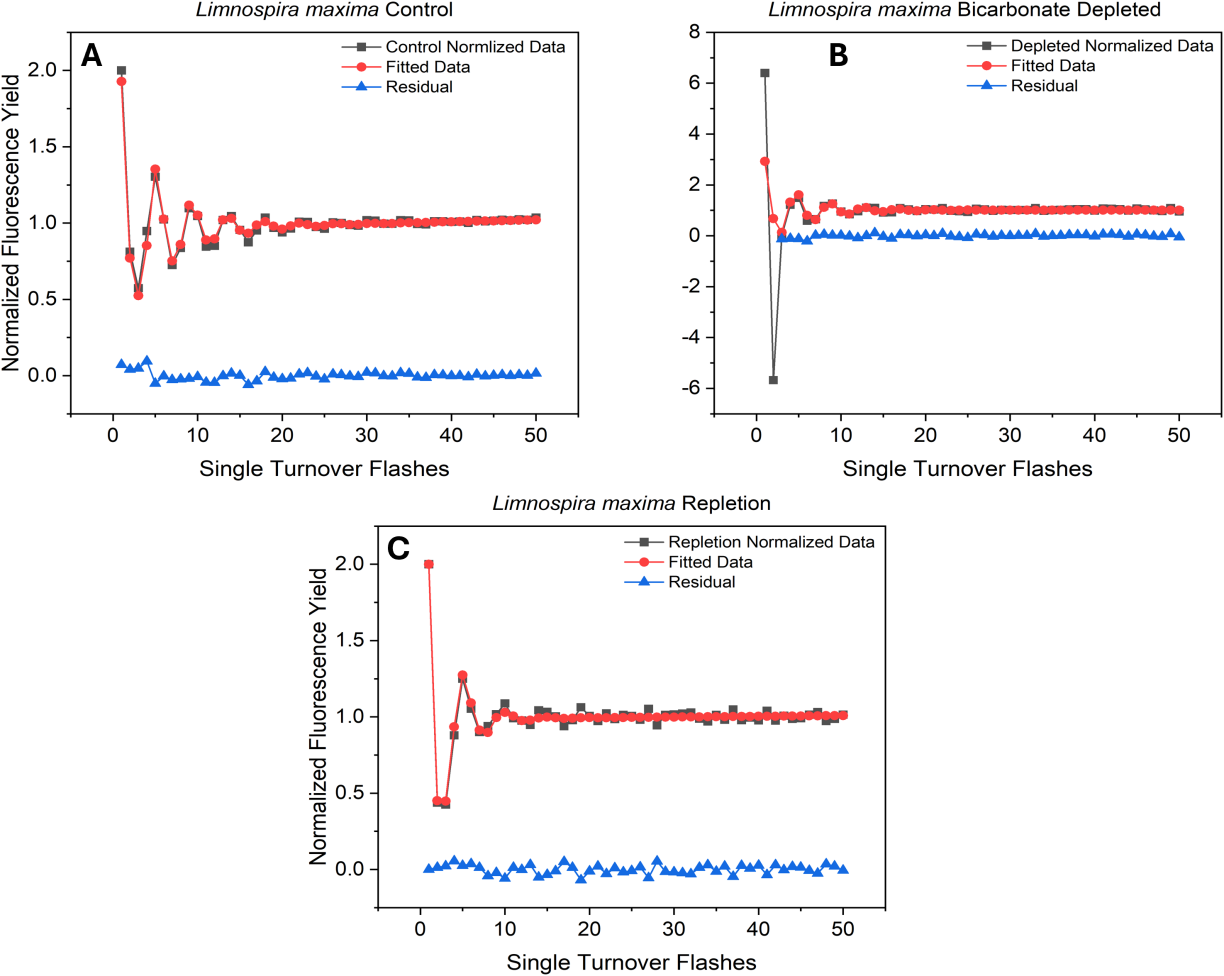
Flash-induced oscillations in F_v_/F_m_ from **(A)** untreated and **(B)** treated cells and **(C)** bicarbonate repleted cells exposed to a train of 50 saturating pulses (STF).

Fluorescence emission from reaction center Chl *a* molecules (P680) within PSII is due to failure to advance electrons to the semi-stable acceptor Q_A_ (Govindjee, 1995). The F_v_/F_m_ values were generated and plotted for the control, bicarbonate depletion, and reversal for *L. maxima* (**Figures 2A–C**) and inefficiency parameters and relative S-state populations were generated using VZAD (Ananyev and Dismukes, 2005; Vinyard et al., 2013b) and are provided in **Table 2**. In the control (**Figure 2A**), as expected, period four oscillations occur, confirming the successful separation of the water oxidizing complex into different S-states and maintenance of separation over time. However, in the depleted *L. maxima* cells (**Figure 2B**), the quality of oscillations is diminished compared to the control. Visible oscillations remain in the depleted cells at much lower intensity, although there is clearly a scaling change after the second flash. Notably, the fit was applied only to flashes from the third onward, with the first two flashes modeled to reflect expected behavior, consistent with the rest of the flash train. In **Figure 2C**, reversing the process of depletion by reintroducing bicarbonate partially restores the oscillatory pattern, producing a close overlap with the control and resolving the two-flash irregularity observed under depletion, though differences in the oscillation profile remain.

**Table 2:** Inefficiency and S-state populations obtained from VZAD model from untreated, treated and bicarbonate repletion cells.

Inefficiency Parameters and S-state Populations								
	α	β	δ	S_0_	S_1_	S_2_	S_3_	RMSE
Control	0.13	0.05	0.00	0.29	0.38	0.33	0.00	2.70%
Depleted	0.11	0.10	0.00	0.15	0.52	0.33	0.00	6.10%
Repletion	0.20	0.00	0.00	0.00	0.43	0.57	0.00	2.98%

The overlap in the F_v_/F_m_ values are nearly identical, though the Kok alpha (failure to advance electrons) parameter is 0.13 for the control, while the reversal has a higher value of 0.20. The depleted cells yielded an alpha value closer to the control (0.11). The delta parameter, indicating the likelihood of electrons returning to the water-oxidizing complex, was zero for all three conditions, as anticipated. This result is expected, given that *L. maxima* is known to lack PSII-cyclic electron flow (Ananyev et al., 2016), and the delta parameter corresponds to this process. Beta, which corresponds to an advance of two oxidation states, was 0.05 for the control and 0.10 in the depleted condition. Repletion, like the control, resulted in a beta parameter of 0. While the S-state populations cannot be definitively determined fluorometrically using a Kok-type model to date (Ananyev and Dismukes, 2005), the relative change in initial S-state distribution can be observed. Interestingly, both depletion and restoration of bicarbonate show shifts to more oxidized S-state balances, although this is clearly affected by depletion rendering some centers inactive after two flashes (**Figure 2B**), which is not fully restored by repletion in **Figure 2C**. The oxidized S-state levels seen are implausibly high and stem from using a model designed to expect product (oxygen) on only one oxidation state when product (fluorescence) is generated from all four states, but the overall change is observable in the raw data.

### 3.4 Q_A_^-^ reoxidation kinetics

**Figure 3:**
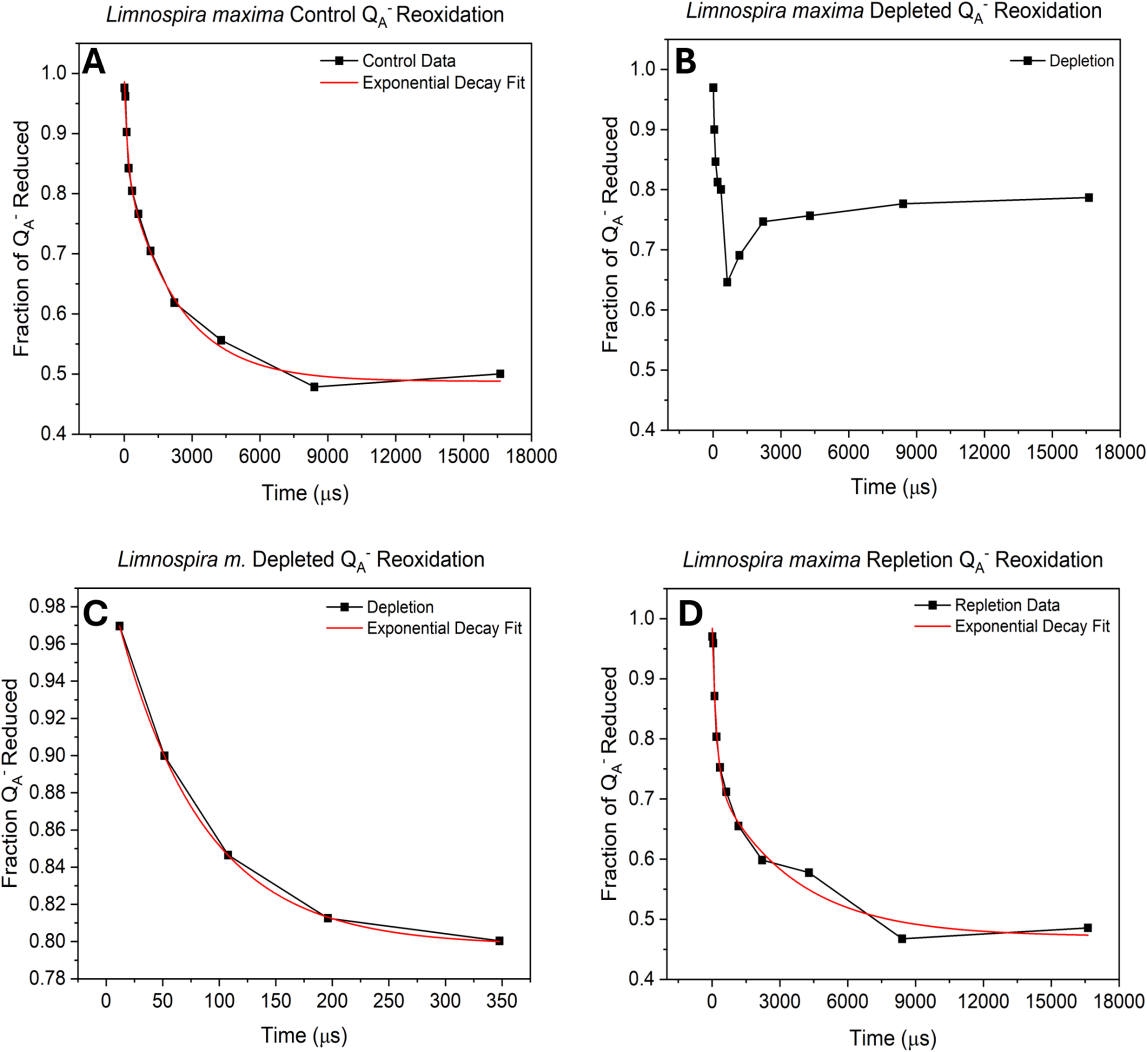
Biphasic exponential decay curve of Q_A_^-^ reoxidation showing **(A)** untreated cells, **(B)** bicarbonate depleted, **(C)** Exponential decay curve of the first 5 flashes in bicarbonate depletion and **(D)** after bicarbonate repletion. All results were obtained using variable-time pump-probe flashes and measuring F_v_/Fm.

The role of bicarbonate, which is coordinated to the non-heme iron between the plastoquinone acceptors Q_A_ and Q_B_ on the acceptor side of PSII, has been extensively studied. Therefore, it was of interest to quantify the electron transfer rate to the PQ pool on the acceptor side and thus the redox poise and occupancy of the Q_B_ site, which is expected to be altered by availability of this specific bicarbonate. Following photochemical charge separation, electrons are transferred from P680 to pheophytin and then rapidly to the nonexchangeable Q_A_, serving as a one-electron transfer to exchangeable Q_B_, the terminal electron acceptor within PSII. Q_B_ accepts two electrons from Q_A_ and incorporates two protons to form PQH_2_ which exchanges into the PQ/PQH_2_ pool, subsequently delivering electrons to cyt b_6_f (Fantuzzi et al., 2022). Reoxidation kinetics of Q_A_^-^ were assessed by saturating flash chlorophyll fluorometry. In **Figure 3A** and **Table 3**, for wild type *L. maxima*, the time it takes to transfer electrons from Q_A_^-^ to Q_B_ is 144 µs and the fraction of PSIIs with Q_B_ in this oxidation state are 0.160 (or 16%); see **Table 3**. The time it takes to transfer an electron to Q_B_^-^ from Q_A_^-^ is 2350 µs and fraction of centers is 0.350 or 35%. In native *L. maxima* the fraction of centers that are inactive or unoccupied is 0.490, which is typical for cyanobacteria (Gates et al., 2016; Vinyard et al., 2019; Gates et al., 2023b).

**Table 3:** Parameters obtained from Q_A_^-^ reoxidation: Y_o_ is centers not active, t_1_ and A_1_ are time for electron to transfer from Q_A_^-^ to Q_B_ and fraction of centers undergoing this transition, respectively; t_2_ and A_2_ are the equivalent parameters for transfer or electrons from Q_A_^-^ to Q_B_^-^.

Q_A_^-^ Reoxidation Parameters			
Parameter	Control	Repletion	Depletion-5 Flashes
Y_o_	0.516 ± 0.03	0.568 ± 0.02	0.800 ± 4.67E-4
t_1_, µs	137 ± 44.5	153 ± 59.7	75.8 ± 0.684
A_1_	0.150 ± 0.02	0.213± 0.06	0.200 ± 6.82E-4
t_2_, µs	1970 ± 598	1230 ± 651	
A_2_	0.330 ± 0.02	0.220 ± 0.06	

t_1_ and t_2_ represent time intervals in microseconds, and A_1_ and A_2_ are corresponding amplitudes, expressed as fractions of 1.

Under depletion (**Figure 3B**), the data points could not be fit to a biphasic exponential curve. Replicate measurements consistently show exponential decay, ceasing after the sixth point (essentially, after the fast phase). Electron transfer proceeds normally until it can no longer transfer to Q_B_^-^. This suggests that without bicarbonate, the plastoquinone pool cannot perform turnovers, indicating an acceptor-side limitation. In **Figure 3C**, the first five data points were fitted, yielding a t_1_ value comparable to the control, indicating a fast kinetic of electron transfer to Q_B_. The Q_A_^-^ reoxidation kinetics were also measured for the repletion of bicarbonate to native *L. maxima* to see if the depletion method was harmful to the operation of PSII (see **Figure 3D** and **Table 3**). In the repletion of *L. maxima*, the time that it takes to make Q_B_^-^ from Q_A_^-^ is 153 µs and the fraction of Q_A_^-^ reduced is 0.213. This suggests that more electrons are going to Q_B_ proportionally. It takes 11% longer for the repleted *L. maxima* (compared to the control, 137 µs) to transfer an electron to Q_B_; however, the fraction of Q_A_^-^ reduced is less in the control (0.150). For the transfer of electrons to Q_B_^-^, the repleted cells take noticeably less time (1230 µs) compared to the control (1970 µs) and the fraction of Q_A_^-^ reduced is different between the two conditions (control: 0.330 and repleted: 0.220).

### 3.5 Cytochrome b_6_f redox kinetics

The cytochrome b_6_f complex, located between PSII and PSI, facilitates electron transfer by oxidizing plastoquinol and reducing plastocyanin (PC) or cytochrome c_6_. It comprises four redox centers: the iron- sulfur cluster (Fe_2_S_2_) and three hemes of cyt b_6_ and cyt f (Baniulis et al., 2008; Tikhonov, 2014). Cytochrome b and f exhibit similar absorption characteristics, with an initial drop (trough) upon illumination, signifying electrons being pulled by the PSI (P700^+^) reaction center from cyt b (or cyt f) through the PC pool. This trough indicates the time needed to remove electrons from cyt b/f and the redox poise, indicating the quantity of electrons transferred. The peak following the trough corresponds to the arrival of electrons from PSII. Subsequently, absorption declines as PSI draws off electrons faster than PSII can replenish them until the light is turned off, restoring redox poise to its dark state equilibrium. For PC, absorption decreases with reduction and increases with oxidation. Initially, absorption increases when P700^+^ oxidizes the PC pool upon illumination. Subsequently, absorption decreases as electrons shuttle from PSII through cyt f (Ungerer et al., 2018).

**Figure 4:**
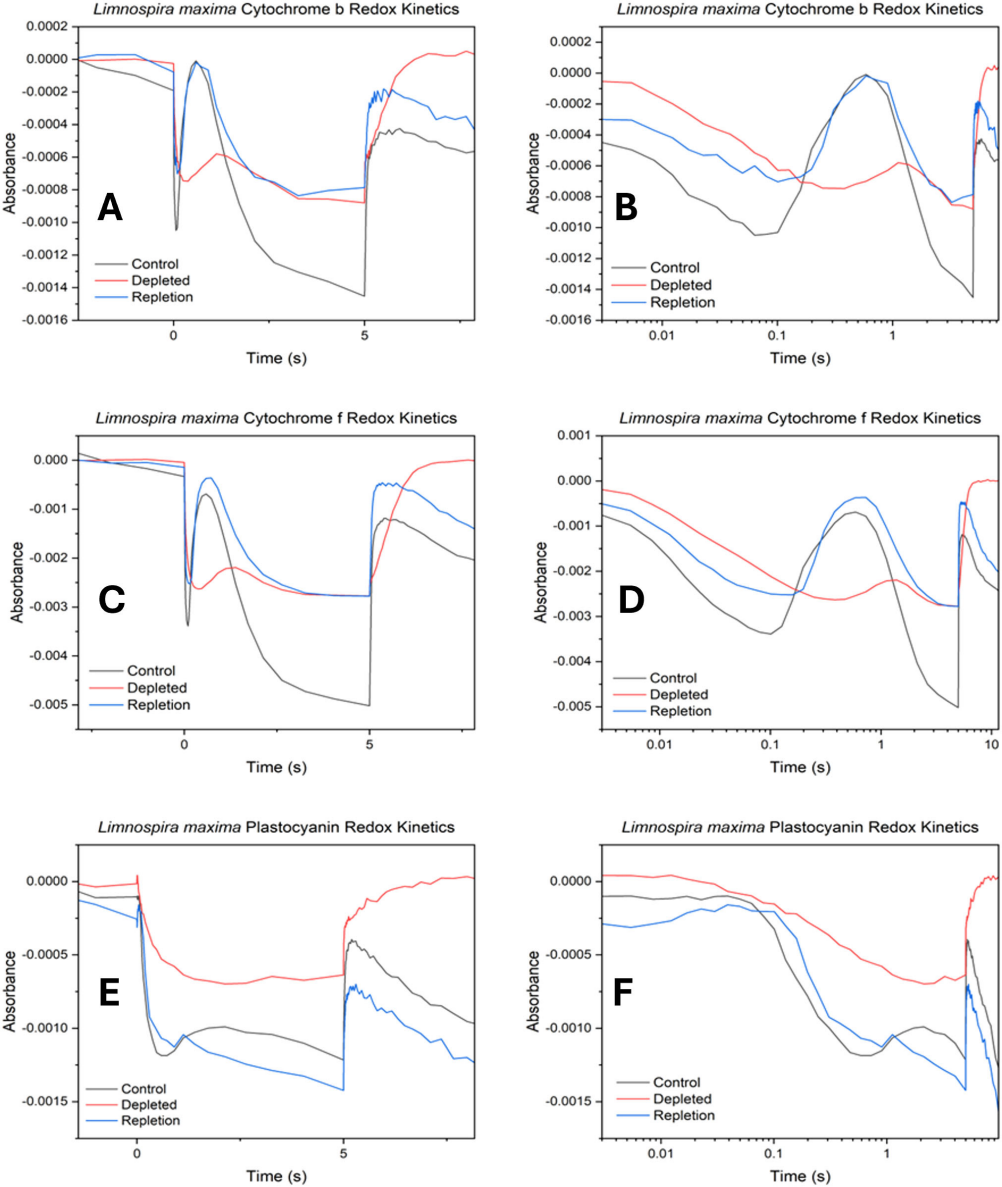
Cytochrome b_6_f oxidation-reduction kinetics in control, depleted and repleted cells. **(A)** cytochrome b kinetics, **(B)** log scale cytochrome b kinetics, **(C)** cytochrome f kinetics **(D)** log scale cytochrome f kinetics, **(E)** plastocyanin kinetics and **(F)** log scale plastocyanin kinetics. The absorption profiles observed for cytochrome b_6_f exhibit the following trends: increasingly negative absorption values signify higher levels of oxidation in hemes b and f, while more positive absorption values indicate reduction.

In **Figure 4A**, the control displays both a rapid extraction of electrons from cyt b, seen by the stronger negative absorption and faster timescale. This is followed by a rise in absorbance, corresponding to reduction, from the resupply of electrons from PSII. This trend is clearly visible on a logarithmic scale in **Figure 4B**. In cyt f (**Figures 4C, D**) measurements, the control shows more negative absorption (greater oxidation), and faster reduction, indicating more electron transfer from PSII overall when bicarbonate is present. However, in the absence of bicarbonate, there is a decrease in the quantity of electrons pulled by PSI and a slower rate of resupply from PSII from cyt b and f. Subsequent observations of cyt b and cyt f following the reduction from PSII suggest that in the absence of bicarbonate, electrons are not replenished as efficiently as in native *L. maxima*. In PC (**Figures 4E, F**), the control experiences less initial oxidation by PSI compared to the depleted sample. In the absence of bicarbonate, PC ultimately becomes more oxidized by PSI seen by the initial feature followed by less resupply of electrons by PSII compared to the control. For repletion, it is observed that cyt b becomes less oxidized after bicarbonate reintroduction, resembling its state during depletion, despite both photosystems functioning normally. This suggests that reversal does not significantly affect electron transfer in cyt b. For PC, the initial kinetic feature for reversal suggests that PSI is taking longer to consume electrons, compared to untreated *L. maxima*.

### 3.6 P700 redox kinetics

**Figure 5:**
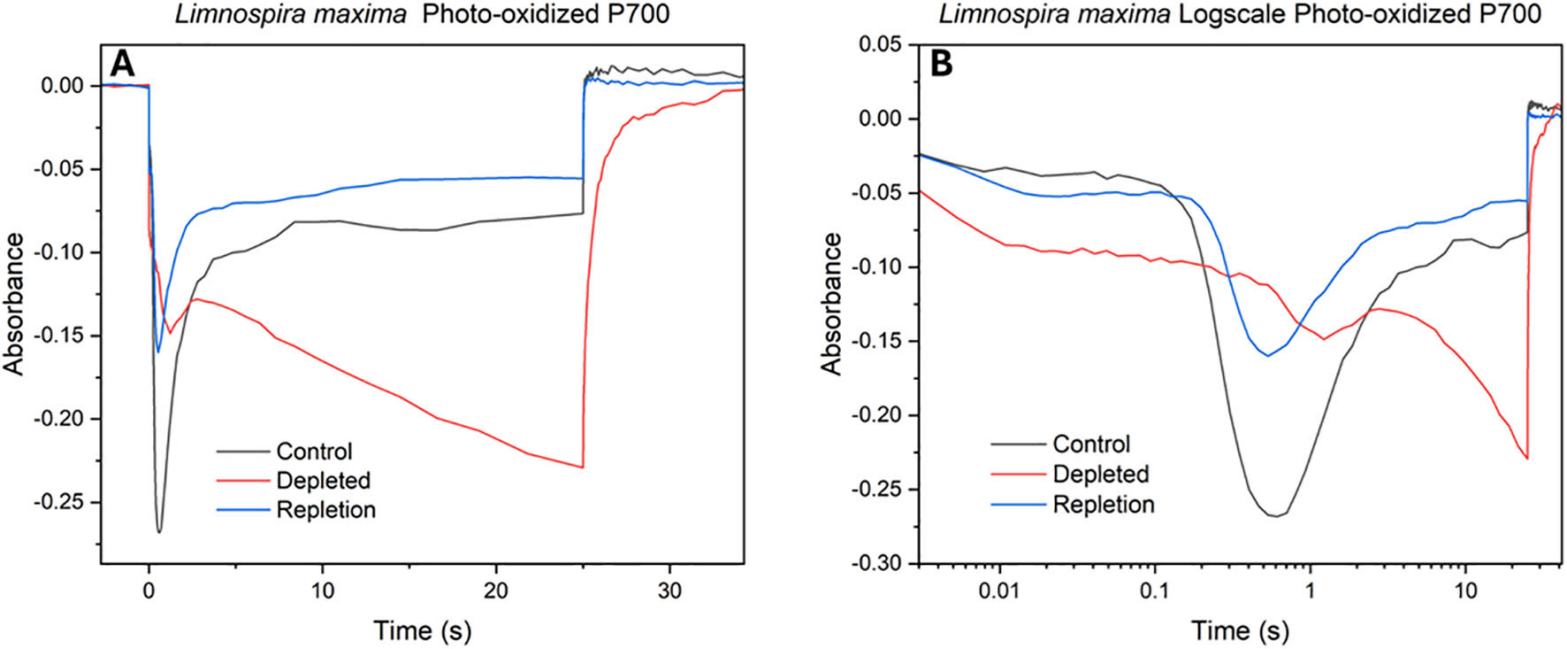
Photooxidized P700^+^ absorbance measurements at 810 nm of **(A)** control, depleted and repleted cells; **(B)** log scale kinetics of the same.

The impact of bicarbonate absence on the electron transport chain concerning the reduction rate of photooxidized P700 (P700^+^) at PSI was observed. Similar to cyt b_6_f redox kinetics, the decrease (negative) in absorption signifies oxidation, while an increase (positive) in absorption corresponds to reduction. The initial feature in redox kinetics corresponds to the consumption of electrons by PSI from plastocyanin, followed by a secondary feature resulting from the delivery of electrons from other carriers higher up in the electron transport chain and ultimately from PSII. In **Figure 5A**, upon illumination a strong initial feature of plastocyanin oxidation by PSI is seen, followed by a secondary feature of quick delivery of electrons from PSII for the control. In bicarbonate-depleted cells, the absence of bicarbonate affects the oxidation of plastocyanin significantly. This is followed by a small secondary feature (peak) due to a limited delivery of electrons from PSII. However, further oxidation occurs with difficulty due to a severe limitation in additional electron supply caused by the lack of bicarbonate, suggesting potential challenges for Rubisco. Normally, as seen in native *L. maxima*, PSI rapidly empties the PC pool, which is then followed by a peak from reduction from PSII activation. However, in the depleted cells PSII activation is delayed and Rubisco activation overwhelms PSI. The delay kinetic feature of electron transfer being slowed down could be due to maximum oxidation achieved prior to electron delivery from PSII. The reversal and control traces exhibit comparable features, showing the reversal is effective both upstream and downstream. Both trend lines plateau similarly at various points, indicating that the bicarbonate reversal affects PSI at a largely consistent rate and the return of electrons from PSII is normal. For the reversal, there is slightly less oxidation by PSI compared to the control.

## 4 Discussion

### 4.1 Fluorescence at 77K

Fluorescence measurements at 77K were conducted to determine how bicarbonate influences the functionality of the photosystems by modulating the photosystem stoichiometry and antenna attachment, and the impact on the two bicarbonate binding sites. Photosystem balancing is imperative for regulating the energetic input from light. Initial charge separation occurs exclusively at PSII, leading to the production of NADPH, while PSI can be used for either linear or cyclic pathways and is more associated with ATP production. The loss of bicarbonate results in higher fluorescence emission of F685 while the control has a lower emission of F685 and higher F695. The F695 emission peak is generated when significantly redshifted chlorophyll molecules associated with the CP47 subunit in PSII re-emit light energy they absorbed; this is only feasible if excitons cannot be transferred to the reaction center, P680 (Andrizhiyevskaya et al., 2005). The ratio of “healthy” (F685) to “unhealthy” (F695) PSII suggests that excitons are not reaching the reaction center in the control as frequently as when there is loss of bicarbonate. Lower fluorescence emission from PSII without bicarbonate indicates reduced chlorophyll connectivity to the PSII reaction center. When bicarbonate is present, chlorophyll connectivity to PSII increases. Excitons reach the reaction center despite greater chlorophyll connectivity to PSI in the absence of bicarbonate. In the control, there are unhealthier PSII on average but higher chlorophyll connectivity.

By measuring emission from the excitation of phycobilin, the balance of antenna exciton usage can be assessed. The F685:F695 ratio for all three conditions differs from that of the chlorophyll excitation. In the presence of bicarbonate, the F685:F695 emission ratio is not significantly lower compared to its absence. The PSII: PSI emission ratio for the control is slightly lower in the presence of bicarbonate. This indicates that bicarbonate loss does not significantly affect PSII antenna attachment or disrupt PSII reaction center fluorescence. The phycobilisome does not appear to dissociate from PSII; it remains proportionally attached to functional PSII even under bicarbonate loss. Phycobiliproteins play a fundamental role in capturing light energy and transferring it to the reaction centers: CPC broadens the range of light that can be harvested for photosynthesis, enhancing light energy capture efficiency, while APC ensures the transfer of absorbed energy to the reaction enters of PSII and PSI. APC fluorescence emission is higher in the absence of bicarbonate compared to when bicarbonate is present. The higher ratio suggests a reduction in the number of excitons being transferred from the antenna to the reaction center and APC is likely the terminal acceptor because when excitons reach APC, they are transferred to PSII if it is coupled (Mullineaux, 2008). In the control, the elevated CPC fluorescence could indicate two possibilities: considerable backup, leading to increased APC, or the presence of free rods, which are regarded as having a shading effect (Tamary et al., 2012). Loss of bicarbonate increases free phycobilin, while its presence reduces free phycobilin. It has been postulated that bicarbonate may be involved in phycobilisome association (Joshua et al., 2005). Phycobilisomes exhibit rapid diffusion on the membrane surface but become immobilized when cells are exposed to high osmotic strength buffers. Prior research has determined that reactions between phycobilisomes and reaction centers are stabilized in the presence of 1 M phosphate buffer, leading to irreversible formation of quenched states (Joshua et al., 2005). The F685 fluorescence emission decrease for depletion indicates that the observed PSII complexes continue to be able to transfer excitons to the reaction center. The overall fluorescence emission band increases with the loss of bicarbonate, which may be attributed to the inhibition of electron transport on the acceptor side. The electrons are being transferred to Q_A_^-^ and do not advance beyond Q_B_^-^, as observed in FRR and Q_A_^-^ reoxidation studies (**Figures 2**, **3**). While the altered F685/F695 fluorescence ratio may suggest partial changes in energy transfer between the antenna and the PSII reaction center, it is also possible that bicarbonate depletion enhances back reaction processes, leading to increased charge recombination from Q_A_^-^. This increased recombination could contribute to both energy dissipation and to reversible dissociation of phycobilisomes from PSII under stress conditions (Joshua and Mullineaux, 2004; Yang et al., 2009; Tamary et al., 2012). Thus, the observed fluorescence changes may reflect a combination of antenna reorganization and altered electron recombination dynamics at PSII. This inhibition of downstream electron transfer leads to charge recombination, resulting in higher fluorescence yield compared to the control.

### 4.2 Fast repetition rate fluorometry

The diminished F_v_/F_m_ oscillation amplitudes when bicarbonate is lost suggest that a fraction of the population of centers are active, and that the functionality of the water oxidizing complex remains in these centers. However, only one or two electrons are released from the WOCs of most, but not all, PSII, indicated by all following flashes resulting in separately fittable oscillations. One of the known roles of bicarbonate on the acceptor side is facilitating electron transfer between (plastoquinone) Q_A_ to Q_B_ (and thus to the PQ pool) (Shevela et al., 2012). Consequently, this leads to the observed two-electron feature in one of the two distinct populations as an effect of depletion; most centers should be inactive, being depleted. These findings suggest that, in the absence of bicarbonate, the transfer of more than two electrons is inhibited. This was initially thought to correspond to an oxidized iron(III) at the NHI site, but under bicarbonate depletion with 100 μM DCMU only one electron is advanced (**Supplementary Figure S8**). DCMU only blocks transfer of electrons from Q_A_ to Q_B_, so based on the FRR data, in which a consistent feature is observed where two flashes are seen, it appears that one electron is going from Q_A_ to Q_B_, forming the semiquinone Q_B_^-^. Q_B_^-^ is retained on a timescale for at least seconds but less than two minutes, as recovery is not observed during a 5s flash train in any significant quantity but the two minute dark acclimation between flash trains is adequate to fully restore this feature. This timescale is consistent with an internal recombination process, via either cyt b_559_ (Shinopoulos and Brudvig, 2012) or Q_c_ (Gates et al., 2023a).

### 4.3 Q_A_^-^ Reoxidation kinetics

The known acceptor side active site of bicarbonate is found coordinated to NHI in between the two plastoquinones Q_A_ and Q_B_. The loss of bicarbonate results in exponential decay ceasing at about 350 microseconds. Electron transfer appears to proceed normally from Q_A_^-^ → Q_B_ but ultimately electrons are obstructed during transfer to reduced Q_B_^-^. As a result, this process hinders the removal of PQH_2_, effectively reducing the removal rate to slower than the timescale measured. Bicarbonate depletion leads to an acceptor side limitation at the secondary quinone acceptor Q_B_, probably due to hindered proton delivery (Kato et al., 2016). The existing fast kinetic during the first phase indicates that under depletion conditions there is electron transfer to Q_B_ forming Q_B_^-^. This implies that quinol formation at the Q_B_ site in this organism occurs via sequential addition of one electron, one proton, and then a second electron and proton only after delivery of the first proton (Sugo and Ishikita, 2022). It has been previously seen that formate depletion of bicarbonate in *Synechocystis* sp. PCC 6803 PSII inhibited proton delivery to plastoquinol (Q_B_H^-^) (Cao et al., 1991). However, in *L. maxima*, only one electron appears to be transferred, and inhibition seems to occur at an earlier step, which suggests a different mechanism than the D1-S268-mediated proton delivery to Q_B_H^-^ seen by Forsman et al (Forsman and Eaton-Rye, 2020). Inhibition could result from lack of proton input from other mechanisms, halting at Q_B_^-^ without proton transfer or stopping delivery of the second electron due to requirement of a concurrent proton transfer which does not occur. Since the first electron transfer is observed, two possible sites of formate depletion must be considered, which are located respectively at the non-heme iron (and possibly affecting D1-H215), and closer to the D1 stromal surface near D1-S264 and D1-H252 (Kato and Noguchi, 2022). S264 and H252 are likely responsible for transfer of the first proton, while H215 facilitates delivery of the second (Kato and Noguchi, 2022). Therefore, if a proton cannot be associated to Q_B_^-^, the site being depleted is probably near H252 and relatively distant from the non-heme iron but near the stromal surface, whereas if a proton can be associated, the site being depleted is most likely the non-heme iron site. The D1-Y246 residue also plays a critical role by facilitating a hydrogen bond (H-bond) network connecting the bicarbonate ligand to the Q_B_ binding pocket by donating an H-bond to bicarbonate and accepting one from a nearby water molecule (Forsman et al., 2019). This linkage integrates the reprotonation pathway from the protein bulk surface near D1-H252 through D1-H215 with the bicarbonate binding site, potentially explaining why formate substitution disrupts PQH_2_ exchange (Sedoud et al., 2011). The proximity of D1-Y246 to the bicarbonate site suggests that its role could also be impacted by bicarbonate depletion (Sugo et al., 2022), further emphasizing the complexity of proton transfer dynamics in this system. We postulate that the limiting step is proton delivery, as there is evidence to suggest that Q_B_ follows an alternating scheme of one electron, one proton, one electron, one proton (Kato and Noguchi, 2022; Hussein et al., 2024) and any limitation of the second electron transfer would be expected to also impede the first, but the opposite effect is observed here.

### 4.4 Absorbance spectroscopy redox kinetics

Results suggest that *L. maxima* can deliver electrons to the cytochromes from its PQ pool shortly after light exposure (peak delivery approximately ~1 s). However, these electrons are swiftly consumed by active PSI and not replenished in the absence of bicarbonate, indicating an active PQ pool but a limitation within PSII. Activation of carbon fixation downstream could also explain this feature, as impaired carbon fixation would affect the downstream electron acceptor availability. In repleted cells, cyt b becomes less oxidized after bicarbonate reintroduction, resembling its state during depletion, despite both photosystems functioning normally. This suggests that reversal does not significantly affect electron transfer in cyt b but rather reflects downstream effects on carbon metabolism. The reason behind this could relate to the impact on carbon fixation: bicarbonate depletion limits the supply of reductants for the Calvin cycle and formate, while adjusting the redox poise, does not restore carbon fixation capacity. While the initial phase of oxidation by PSI and reduction by PSII returns to normal, the consistent difference in rate aligns more closely with carbon fixation. This is consistent with the known activation time scale of Rubisco at approximately 3–4 seconds (Ruban, 2015). In cyt f, more electrons are resupplied from PSII after bicarbonate repletion. This is expected to be due to bicarbonate being reintroduced to the acceptor side of PSII and facilitating electron transfer to Q_B_. However, in depleted *L. maxima*, electron delivery from PSII to the cytochrome b_6_f complex is significantly delayed from PSII. In PC, PSII can resupply some electrons slowly but then the PC pool rapidly starts to undergo oxidation again, consistent with limited upstream electron input from PSII.

In bicarbonate depleted cells, the absence of bicarbonate significantly hinders the oxidation of plastocyanin, leading to its prolonged reduced state. Further oxidation occurs with difficulty due to a severe limitation in additional electron supply caused by a lack of bicarbonate, suggesting a disruption in continuous electron flow that may impact downstream processes such as carbon fixation, as the electrons on PC cannot be sourced from PSII. In native *L. maxima*, PSI rapidly consumes electrons that are available to it by drawing down from PC, which is then reduced from PSII activation to resume supplying PSI. However, in the depleted cells PSII activation is delayed and Rubisco activation or PSI-cyclic electron flow could overwhelm PSI, which should not be directly affected by bicarbonate depletion. The delay of electron transfer could suggest maximum oxidation of P700 is achieved prior to electron delivery from PSII. While this study primarily focused on the impact of bicarbonate depletion on PSII electron transport, the novel effects observed on PSI redox kinetics highlight an important new direction for future work. Further studies will be needed to comprehensively investigate the effect of bicarbonate depletion on PSI regulation, including donor side and acceptor side dynamics under physiological stress.

## 5 Conclusion

We confirm the acceptor-side role of the bicarbonate which can be depleted from *L. maxima* PSII most easily. We attribute the specific activity of this bicarbonate site to facilitating the transfer of electrons to Q_B_ and specifically Q_B_^-^. We establish that this bicarbonate depletion method, which has relatively mild effects on photosystem II, also leads to reversible disconnection of the light-harvesting antenna pigments. Beyond PSII, bicarbonate depletion alters cytochrome b_6_f reoxidation kinetics and delays P700 reoxidation kinetics, suggesting impaired carbon fixation and donor side limitation at PSI. These broader effects suggest that bicarbonate is critical for maintaining intersystem electron flow and highlights future directions and considerations for investigation of the other known PSII bicarbonate depletion techniques.

## Data Availability

The raw data supporting the conclusions of this article will be made available by the authors, without undue reservation.
