# Supplementary material for: Depletion reveals role of bicarbonate in the photosynthetic electron transport chain of Limnospira maxima
Source: Front Plant Sci. 2025 Jun 16;16:1584909. doi: 10.3389/fpls.2025.1584909 (PMC12206718; doi:10.3389/fpls.2025.1584909)
Supplement: Supplementary file 1 [file SupplementaryFile1.docx]

**Supporting Information for:** Depletion Reveals Role of Bicarbonate in the Photosynthetic Electron Transport Chain of *Limnospira maxima*

**Authors:** Leslie Castillo^1^, Stavroula Nicolaou^2^, and Colin Gates^1,3^

**Affiliations:** ^1^Department of Chemistry and Biochemistry, ^2^Department of Biology, and ^3^Department of Bioinformatics, Loyola University Chicago, 1068 W. Sheridan Rd. 60660

**Corresponding Author:** Colin Gates, [cgates4@luc.edu](mailto:cgates4@luc.edu)

**
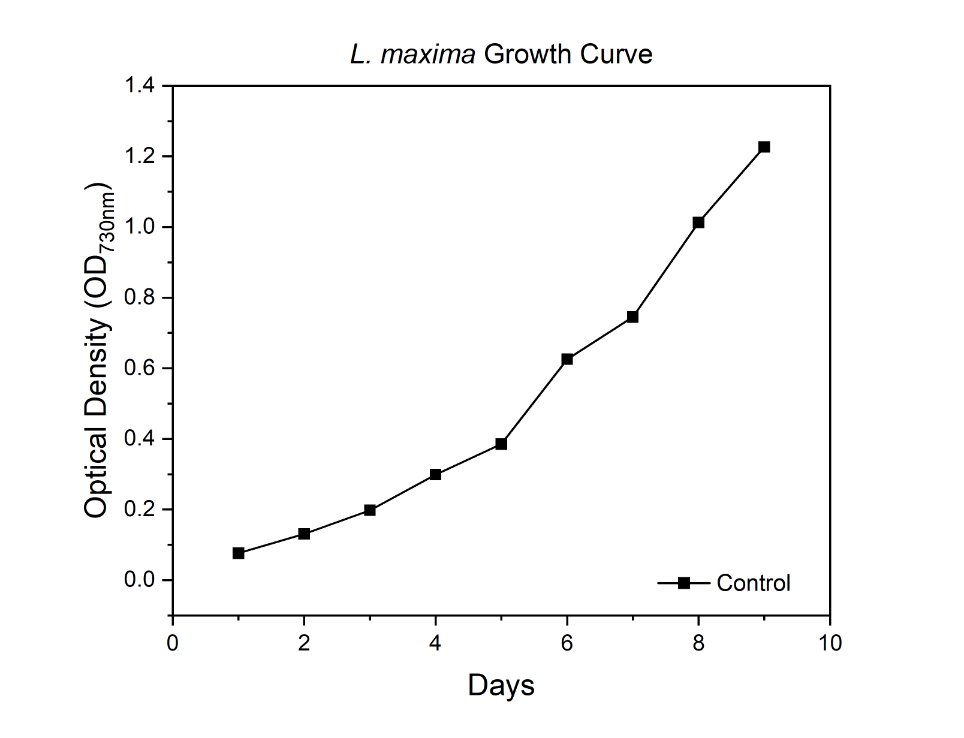
**

**SI Figure 1.** Representative growth curve of native *Limnospira maxima* was measured for a period of nine days by spectrophotometry. The data is representative of three biological replicates.

|  | **Oxygen**  **Consumption** | **Oxygen**  **Evolution** | **Oxygen**  **Production** | **µmol O_2_/mg chl *a*/h** |
| --- | --- | --- | --- | --- |
| **Control** | -0.039 | 0.288 | 0.327 | 164.14 |
| **Depleted** | -0.010 | 0.221 | 0.231 | 69.41 |
| **Repletion** | -0.017 | 0.349 | 0.366 | 165.98 |

**SI Table 1.** Values obtained for oxygen consumption, oxygen evolution and the rate of oxygen production of native, depleted and repleted *L. maxima.*

*.  
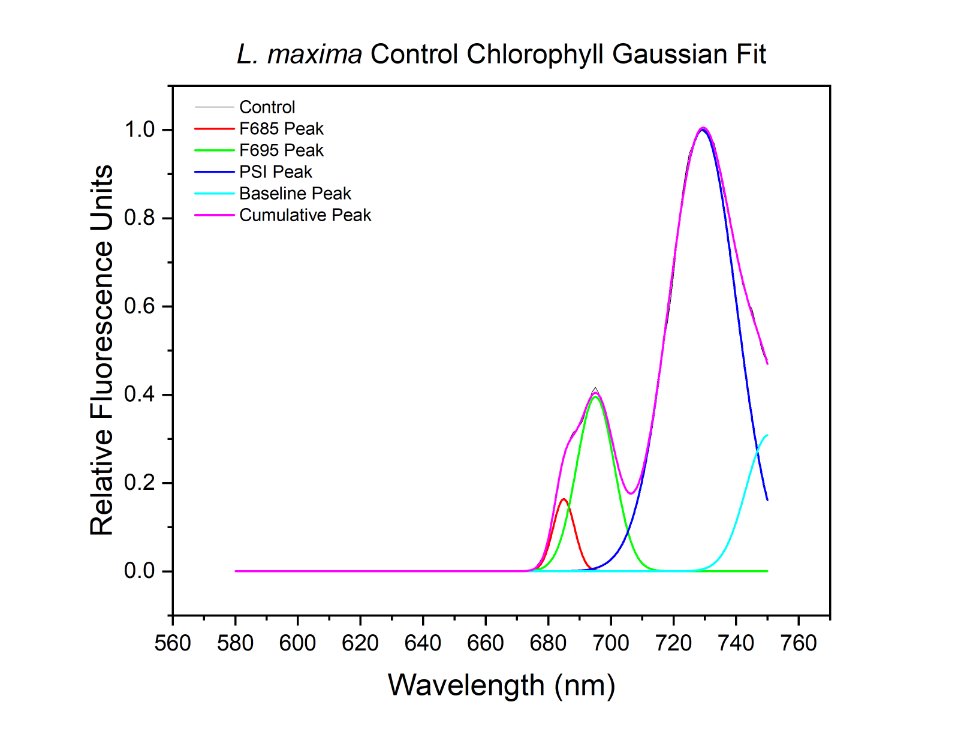
*

**SI Figure 2:** Gaussian fits of chlorophyll fluorescence emission spectra were performed for control *L. maxima*. The areas of the peaks, corresponding from left to right to F685, F695, and PSI, were used to calculate emission ratios at 77K. The PSII emission area was determined as the sum of F685 and F695.

| **Gaussian Fit of Control Chlorophyll Emission at 77K of *L. maxima*** | | | | | | |
| --- | --- | --- | --- | --- | --- | --- |
| Plot | F685 Peak | F695 Peak | PSI Peak | Baseline Peak |  |  |
| Area | 1.40 ± 0.13 | 5.96 ± 0.15 | 27.7 ± 0.33 | 5.68 ± 0.66 |  |  |
| Adj. R-Square | 0.99965 | | | | |  |

**SI Table 2:** Control values for *L. maxima* were determined by applying Gaussian fits to each peak corresponding to the excitation of chlorophyll pigment-protein complexes at 77K. The peak areas were used to calculate the emission ratios.

 
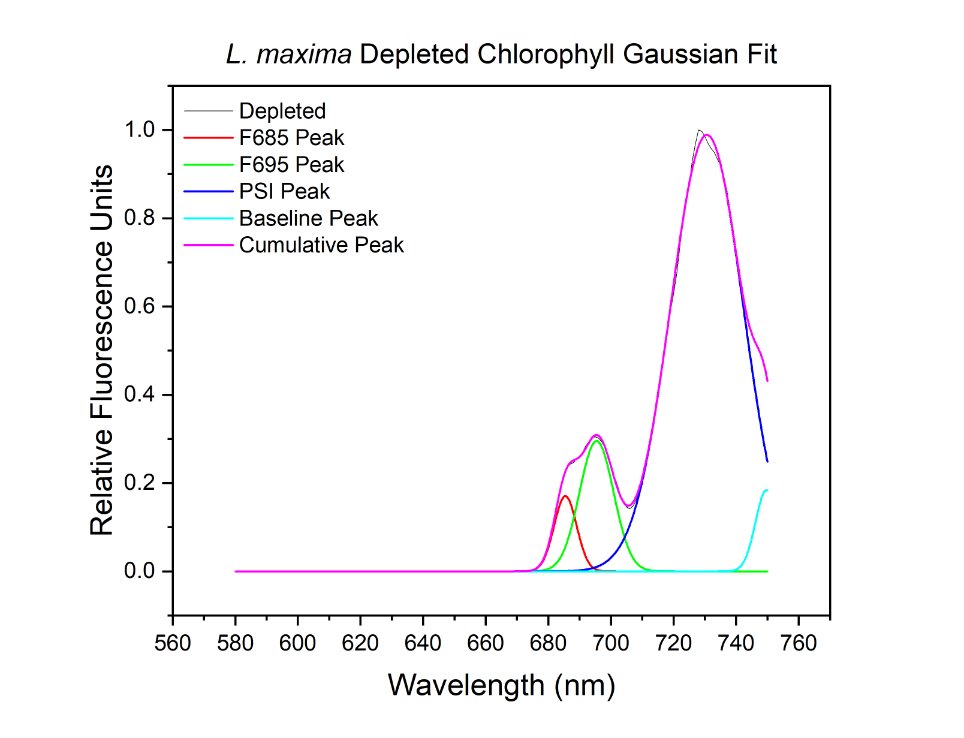


**SI Figure 3:** Gaussian fits of chlorophyll fluorescence emission spectra were performed for depleted *L. maxima*. The areas of the peaks, corresponding from left to right to F685, F695, and PSI, were used to calculate emission ratios at 77K. The PSII emission area was determined as the sum of F685 and F695.

| **Gaussian Fit of Depleted Chlorophyll Emission at 77K of *L. maxima*** | | | | |
| --- | --- | --- | --- | --- |
| Plot | F685 Peak | F695 Peak | PSI Peak | Baseline Peak |
| Area | 1.54 ± 0.15 | 4.04 ± 0.16 | 28.84 ± 0.11 | 1.61 ± 0.18 |
| Adj. R-Square | 0.99948 | | | |

**SI Table 3**: Bicarbonate depletion values for *L. maxima* were determined by applying Gaussian fits to each peak corresponding to the excitation of chlorophyll pigment-protein complexes at 77K. The peak areas were used to calculate the emission ratios.

 
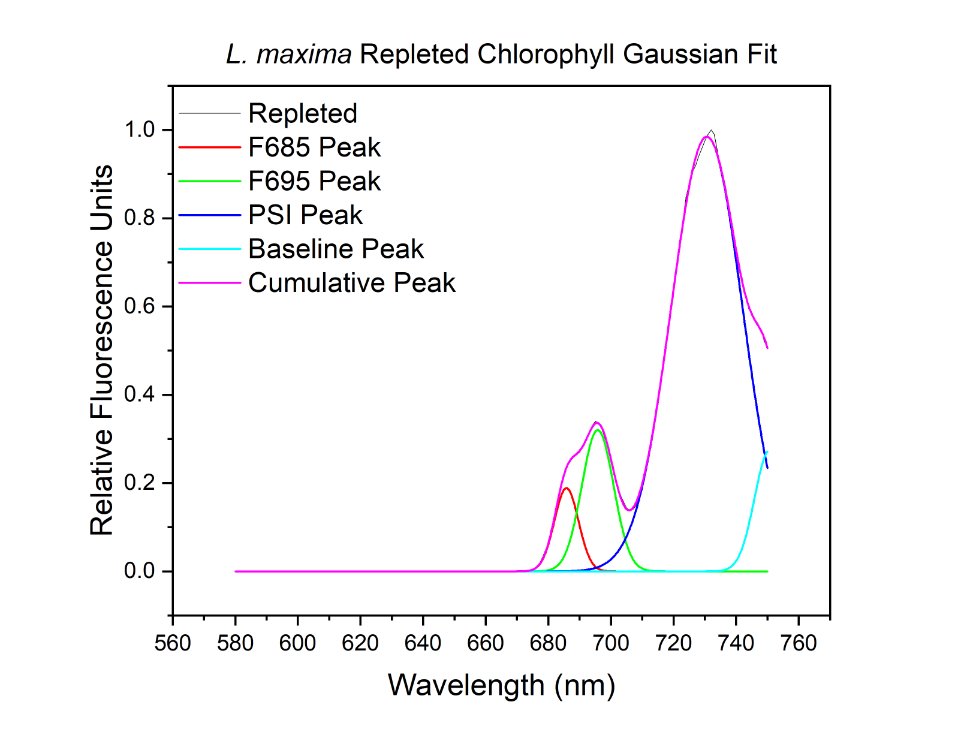


**SI Figure 4:** Gaussian fits of chlorophyll fluorescence emission spectra were performed for repleted *L. maxima*. The areas of the peaks, corresponding from left to right to F685, F695, and PSI, were used to calculate emission ratios at 77K. The PSII emission area was determined as the sum of F685 and F695.

| **Gaussian Fit of Repleted Chlorophyll Emission at 77K of *L. maxima*** | | | | |
| --- | --- | --- | --- | --- |
| Plot | F685 Peak | F695 Peak | PSI Peak | Baseline Peak |
| Area | 1.82 ± 0.12 | 4.08 ± 0.13 | 28.2 ± 0.11 | 3.33 ± 0.30 |
| Adj. R-Square | 0.99971 | | | |

**SI Table 4:** Bicarbonate repletion values for *L. maxima* were determined by applying Gaussian fits to each peak corresponding to the excitation of chlorophyll pigment-protein complexes at 77K. The peak areas were used to calculate the emission ratios.

 
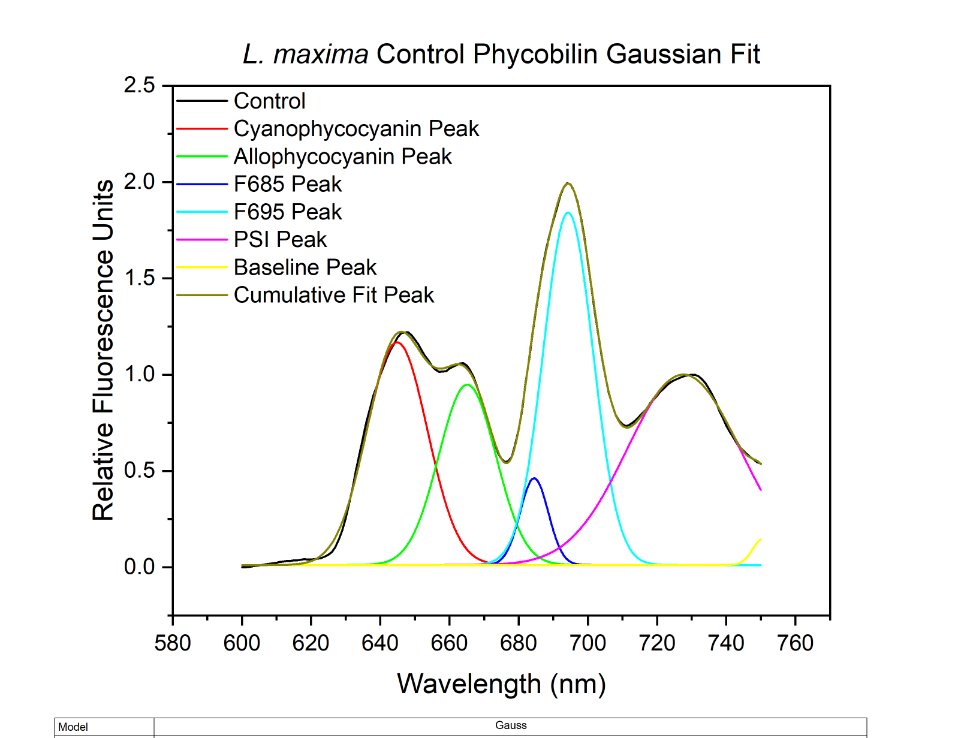


**SI Figure 5:** Gaussian fits of phycobilin fluorescence emission spectra for control *L. maxima*. The areas of the peaks, corresponding from left to right to c-phycocyanin (CPC), allophycocyanin (APC), F685, F695, and PSI, were used to calculate emission ratios at 77K. The PSII emission area was determined as the sum of F685 and F695.

| **Gaussian Fit of Control Phycobilin Emission at 77K of *L. maxima*** | | | | | | |
| --- | --- | --- | --- | --- | --- | --- |
| Plot | CPC Peak | APC Peak | F685 Peak | F695 Peak | PSI Peak | Baseline |
| Area | 25.4 ± 0.74 | 19.4 ± 0.76 | 4.61 ± 1.09 | 33.1 ± 1.36 | 40.6 ± 0.73 | 1.03 ± 0.73 |
| Adj. R-Square | 0.99871 | | | | | |

**SI Table 5:** Control values for *L. maxima* were determined by applying Gaussian fits to each peak corresponding to the excitation of phycobilin pigment-protein complexes at 77K. The peak areas were used to calculate the emission ratios.

 
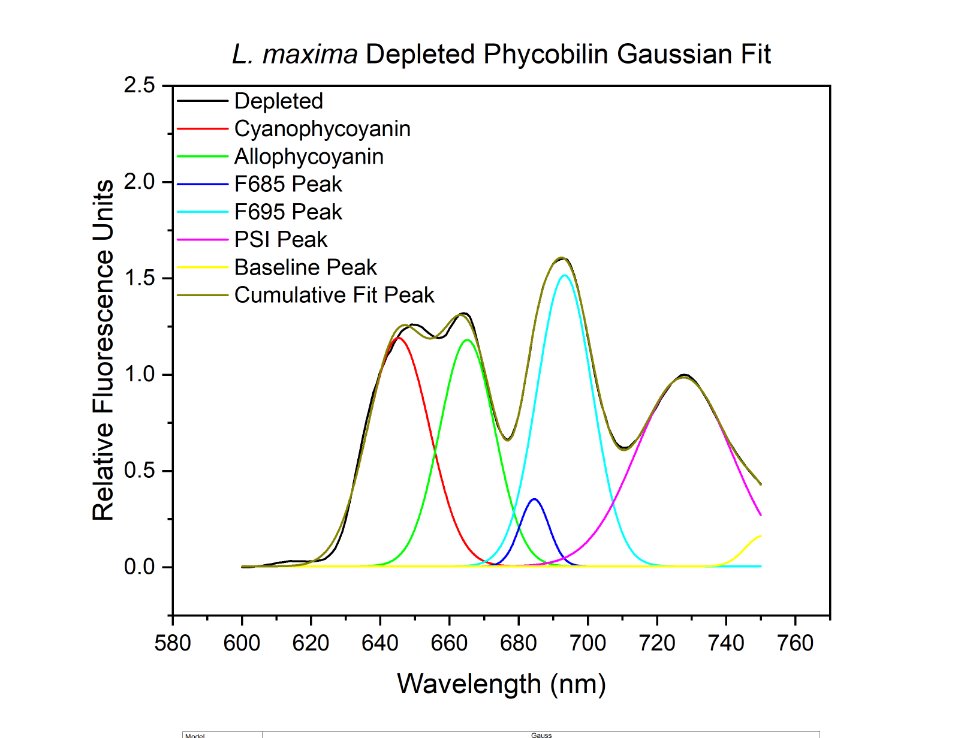


**SI Figure 6:** Gaussian fits of phycobilin fluorescence emission spectra for depleted *L. maxima*. The areas of the peaks, corresponding from left to right to c-phycocyanin (CPC), allophycocyanin (APC), F685, F695, and PSI, were used to calculate emission ratios at 77K. The PSII emission area was determined as the sum of F685 and F695.

| **Gaussian Fit of Depleted Phycobilin Emission at 77K of *L. maxima*** | | | | | | |
| --- | --- | --- | --- | --- | --- | --- |
| Plot | CPC Peak | APC Peak | F685 Peak | F695 Peak | PSI Peak | Baseline |
| Area | 26.7 ± 0.99 | 23.5 ± 1.1 | 3.68 ± 2.28 | 30.3 ± 2.8 | 33.9 ± 1.0 | 2.00 ± 1.4 |
| Adj. R-Square | 0.99781 | | | | | |

**SI Table 6:** Depleted values for *L. maxima* were determined by applying Gaussian fits to each peak corresponding to the excitation of phycobilin pigment-protein complexes at 77K. The peak areas were used to calculate the emission ratios.

 
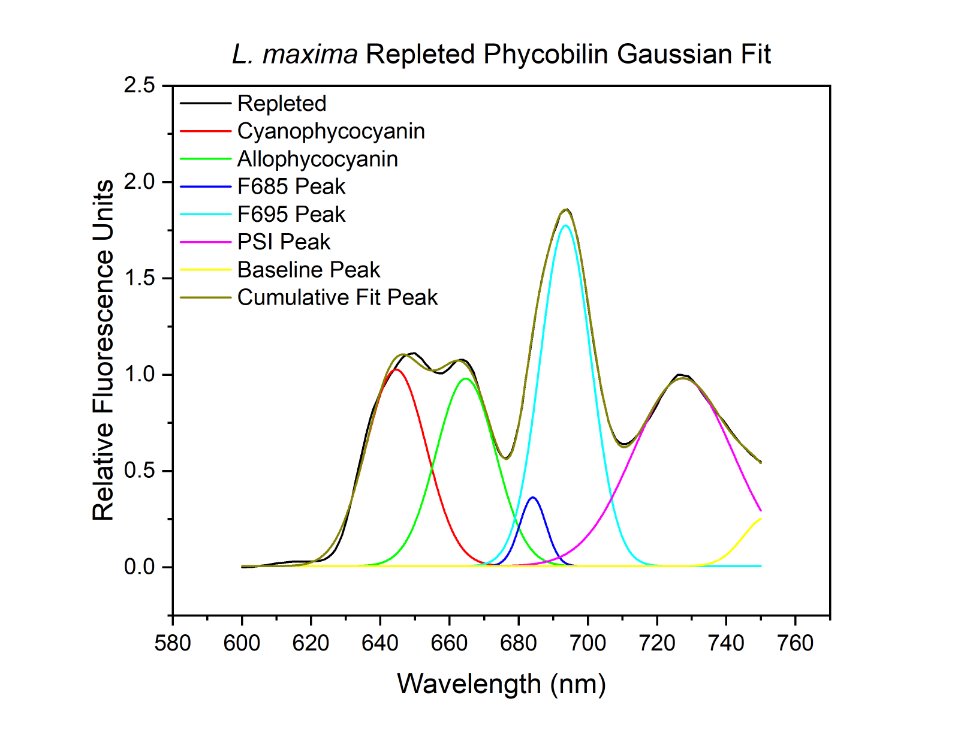


**SI Figure 7:** Gaussian fits of phycobilin fluorescence emission spectra for repleted *L. maxima*. The areas of the peaks, corresponding from left to right to c-phycocyanin (CPC), allophycocyanin (APC), F685, F695, and PSI, were used to calculate emission ratios at 77K. The PSII emission area was determined as the sum of F685 and F695.

| **Gaussian Fit of Repleted Phycobilin Emission at 77K of *L. maxima*** | | | | | | |
| --- | --- | --- | --- | --- | --- | --- |
| Plot | CPC Peak | APC Peak | F685 Peak | F695 Peak | PSI Peak | Baseline |
| Area | 22.4 ± 0.96 | 20.99 ± 1.0 | 3.41 ± 1.2 | 32.8 ± 1.6 | 35.3 ± 1.6 | 4.41 ± 2.8 |
| Adj. R-Square | 0.99815 | | | | | |

**SI Table 7:** Bicarbonate repleted values for *L. maxima* were determined by applying Gaussian fits to each peak corresponding to the excitation of phycobilin pigment-protein complexes at 77K. The peak areas were used to calculate the emission ratios.


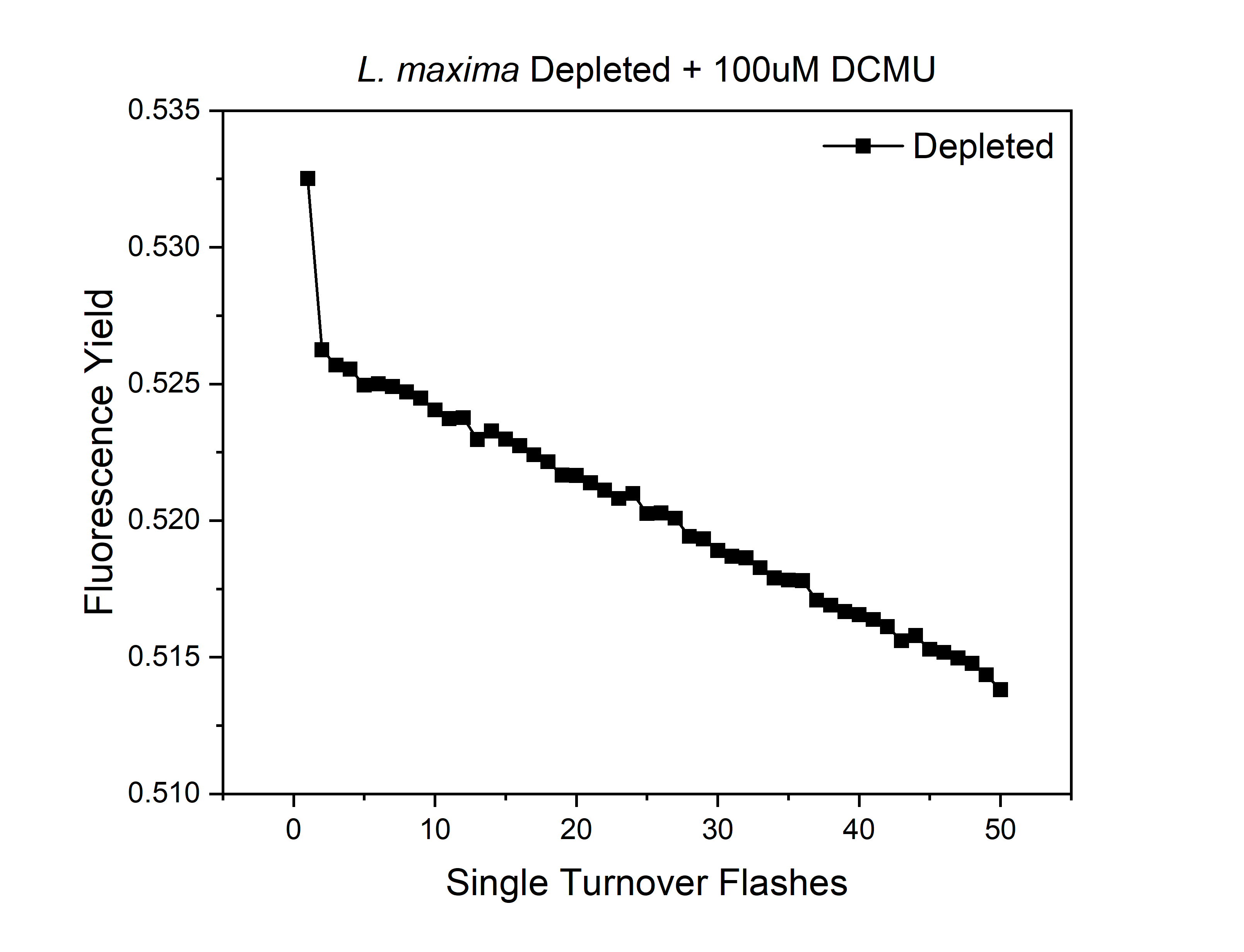


**SI Figure 8.** Fluorescence yield of 50 single-turnover flashes (in F_v_/F_m_) delivered to depleted *Limnospira maxima* treated with 100 μM 3-(3,4-dichlorophenyl)-1,1-dimethylurea (DCMU).
